# Towards scaling Twitter for digital epidemiology of birth defects

**DOI:** 10.1038/s41746-019-0170-5

**Published:** 2019-10-01

**Authors:** Ari Z. Klein, Abeed Sarker, Davy Weissenbacher, Graciela Gonzalez-Hernandez

**Affiliations:** 1 Department of Biostatistics, Epidemiology, and Informatics Perelman School of Medicine University of Pennsylvania, Philadelphia, PA USA; 2 Department of Biomedical Informatics, Emory University School of Medicine, Atlanta, GA USA

**Keywords:** Epidemiology, Data mining

## Abstract

Social media has recently been used to identify and study a small cohort of Twitter users whose pregnancies with birth defect outcomes—the leading cause of infant mortality—could be observed via their publicly available tweets. In this study, we exploit social media on a larger scale by developing natural language processing (NLP) methods to automatically detect, among thousands of users, a cohort of mothers reporting that their child has a birth defect. We used 22,999 annotated tweets to train and evaluate supervised machine learning algorithms—feature-engineered and deep learning-based classifiers—that automatically distinguish tweets referring to the user’s pregnancy outcome from tweets that merely mention birth defects. Because 90% of the tweets merely mention birth defects, we experimented with under-sampling and over-sampling approaches to address this class imbalance. An SVM classifier achieved the best performance for the two positive classes: an F_1_-score of 0.65 for the “defect” class and 0.51 for the “possible defect” class. We deployed the classifier on 20,457 unlabeled tweets that mention birth defects, which helped identify 542 additional users for potential inclusion in our cohort. Contributions of this study include (1) NLP methods for automatically detecting tweets by users reporting their birth defect outcomes, (2) findings that an SVM classifier can outperform a deep neural network-based classifier for highly imbalanced social media data, (3) evidence that automatic classification can be used to identify additional users for potential inclusion in our cohort, and (4) a publicly available corpus for training and evaluating supervised machine learning algorithms.

## Introduction

Despite the fact that birth defects are the leading cause of infant mortality in the United States,^1^ methods for observing pregnancies with birth defect outcomes remain limited (e.g., clinical trials,^2,3^ animal studies,^4^ pregnancy exposure registries^5–8^). Considering that 40% of US adults between ages 18 and 29 use Twitter,^9^ in recent work,^10^ we sought to identify a cohort of women whose pregnancies with birth defect outcomes could be observed via their publicly available tweets. In that work, we identified a small cohort in a database containing the timelines—the publicly available tweets posted by a user over time—of more than 100,000 users automatically identified via their public announcements of pregnancy on Twitter.^11^ Then, we used their timelines to conduct an observational case-control study,^12^ in which we compared select risk factors^13^ among the women reporting a birth defect outcome (cases) and users for whom we did not detect a birth defect outcome, selected from the same database (controls). The study found that reports of medication exposure^14^ were statistically significantly greater among the cases than the controls.

More generally, our recent work demonstrates several valuable ways in which our social media-mining approach could complement existing methods for studying birth defects, most of which continue to have unknown etiologies.^15^ Our pipeline^11^ begins by collecting all the publicly available tweets of women who have announced their pregnancy on Twitter, which enables the use of social media for selecting internal comparator groups, and provides a unique opportunity of exploring unknown risk factors among the chatter. Furthermore, the users we studied were posting tweets not only during pregnancy; most were posting tweets leading up to their pregnancy and before they could have been aware they were pregnant. Thus, our social media-mining approach can be used to observe risk factors in the periconceptional period and early in the first trimester, respectively. Because our pipeline continues to collect the tweets that users post after pregnancy, social media provides a means of long-term follow-up after birth.

While promising, our preliminary studies also demonstrate the limitations of *manual* methods for identifying cohorts on Twitter. Upon grappling with collecting tweets that mention birth defects from among the more than 400 million tweets in our database, we manually annotated ~17,000 retrieved tweets for whether they refer to the user’s birth defect outcome (true positives) or merely mention birth defects (false positives). Then, we analyzed ~650 timelines of the users who posted true positives and identified 195 women for inclusion in our cohort.^10^ This cohort was sufficient for the purpose of studying birth defects in general,^12^ but, as with many pregnancy exposure registries,^16^ it did not include enough specific cases for studying select birth defects, especially those that are more rare, such as encephalocele, biliary atresia, bladder exstrophy, and trisomy 13. However, despite the relatively small cohort initially identified and studied, new users are being constantly added to our database.^11^ In fact, already, the total number of users and tweets in our database has more than doubled since our preliminary studies, so methods capable of automatically identifying cohorts among this growing population are needed in order to exploit social media data for studying birth defects on this larger scale. The development of such methods may be particularly valuable for studying birth defects that are more rare in the general population because of the limited availability of data from traditional sources.

As the first step in automatically identifying cohorts on social media, the primary objective of the present study is to develop methods for automatically detecting tweets by mothers reporting that their child has a birth defect. We took a tweet-level approach to identifying users (as opposed to topic modeling,^17^ for example) because we found that, among the thousands of tweets in users’ timelines, tweets related to their birth defect outcomes appeared to be sparse. Given that the prevalence of birth defect outcomes in the United States is only 3%^18^ (as a comparison, postpartum depression^19^ affects up to 15% of mothers^20^), automatically detecting such rare events on social media is difficult.^21^ In particular, the high degree of class imbalance among tweets that mention birth defects presents challenges for training machine learning algorithms and applying them in practice.^22–24^ Among the 22,999 tweets retrieved using an extensive lexicon of birth defects,^10^ only ~10% of them were actually labeled “defect” or “possible defect” (true positives) by the annotators, and 90% were “non-defect” tweets (false positives). These three classes are summarized as follows:

- *Defect* (+): The tweet refers to the user’s child and indicates that he or she has the birth defect mentioned in the tweet (e.g., *My little miracle, we are so blessed to have you #hypoplasticleftheartsyndrome #hlhs*).
- *Possible Defect* (?): The tweet is ambiguous about whether someone is the user’s child and/or has the birth defect mentioned in the tweet (e.g., *Scheduling an appointment for [name] to confirm a craniosynostosis diagnosis*).
- *Non-defect* (−): The tweet does not refer to someone who is or may be the user’s child and has or may have the birth defect mentioned in the tweet (e.g., *Sally Phillips: My son has Down’s syndrome – but I wouldn’t want to live in a world without it via @[username]*).

In addition to the class imbalance problem, the sample tweets above illustrate further challenges—posed by the text itself—of automatically detecting tweets by mothers reporting that their child has a birth defect, including the various ways users may colloquially refer to their child on social media (e.g., *my little miracle*). The tweet also may not explicitly encode that the user’s child has a birth defect, but rather imply it using nontraditional textual representations afforded by social media (e.g., *#hypoplasticleftheartsyndrome #hlhs*). Even when tweets do contain a common reference to a child (e.g., *my son*) and explicitly state that the child has a birth defect (e.g., *has Down’s syndrome)*, they may be “reported speech”—that is, presenting what was said by others (e.g., *Sally Phillips*). Finally, some tweets do not provide the context on which a full understanding of whether or not the user’s child has a birth defect depends, perhaps because of the character limit imposed by Twitter or the shared background knowledge among users interacting in a social community. For this reason, we created the “possible defect” class, which introduces the challenge of multiclass classification and, moreover, modeling the nuances that distinguish “possible defect” tweets.

In this study, we preprocessed the 22,999 annotated tweets and used them in experiments to train and evaluate supervised machine learning algorithms, including Multinomial Naive Bayes (NB), support vector machine (SVM), and long short-term memory neural network (LSTM) classifiers. In our experiments, we performed various under-sampling and over-sampling methods to address the class imbalance problem. In this paper, we present (1) benchmark results of natural language processing and supervised classification methods, (2) a comparison of feature-engineered and deep learning-based classifiers trained on imbalanced, under-sampled, and over-sampled social media data, (3) the predictions of our automatic pre-filtering, pre-processing, and classification system deployed on ~600 million unlabeled tweets, and (4) an error analysis that could inform strategies for improving classification performance using the training set of 14,716 annotated tweets (Supplementary Data 1) and development set of 3681 annotated tweets (Supplementary Data 2) that we have made publicly available in the supplementary information files.

## Results

### Classification

**Fig. 1:**
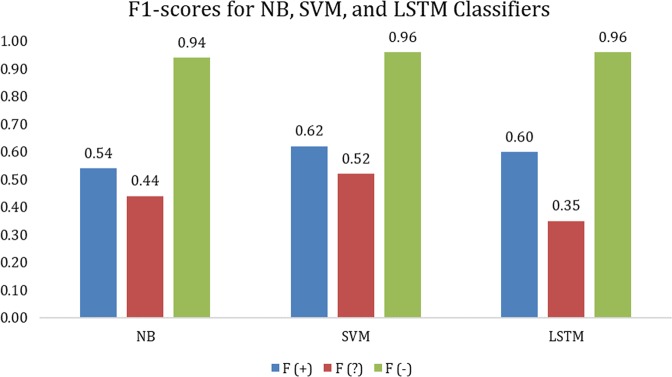
F_1_-scores (F) for “defect” (+), “possible defect” (?), and “non-defect” (−) tweet classes for three classifiers trained on the original, imbalanced data set

The classifiers were evaluated on a held-out test set—a random sample of 20% of the annotated corpus (4602 tweets), stratified based on the natural, imbalanced distribution of “defect,” “possible defect,” and “non-defect” tweets that would be automatically detected by the lexicon-based retrieval^10^ in practice. Figure 1 presents the performance of the NB, SVM, and LSTM classifiers, as measured by F_1_−score, for the “defect,” “possible defect,” and “non-defect” classes:

$$\begin{array}{l}{\rm{F}}_1\,-\,{\rm{score}}\,=\,\frac{2\,x\,{\rm{recall}}\,\times\,{\rm{precision}}}{{\rm{recall}}\,+\,{\rm{precision}}};\,{\rm{recall}}=\frac{{\rm{true}}\,{\rm{positives}}}{{\rm{true}}\,{\rm{positives}}\,+\,{\rm{false}}\,{\rm{negatives}}};\, {\rm{precision}}=\frac{{\rm{true}\,\rm{positives}}}{{\rm{true}\,{\rm{positives}}\,+\,{\rm{false}}\,{\rm{positives}}}}\end{array}$$

In Fig. 1, the classifiers were trained on the original, imbalanced data (14,716 tweets), with only word n-grams (*n* = 1, 2, and 3) as features for the NB and SVM classifiers, and word embeddings trained on tweets for the LSTM classifier. This training set was obtained by splitting the remaining 80% of the 22,999 annotated tweets into 80% (training) and 20% (development) stratified random sets. The SVM and LSTM classifiers outperformed the NB classifier for the “defect” and “non-defect” classes, but the NB classifier outperformed the LSTM classifier for the “possible defect” class. The SVM classifier outperformed the LSTM classifier for the “defect” and “possible defect” classes, and achieved similar performance for the “non-defect” class.

To address the class imbalance problem at the data level, the classifiers were trained on variations of the original, imbalanced data. Table 1 compares the performance of the classifiers when they were trained on the original data with their performance when they were trained using various under-sampling and over-sampling approaches. For the NB classifier, neither under-sampling nor over-sampling improved performance for the “defect” and “possible defect” classes. For the SVM classifier, neither under-sampling nor over-sampling improved performance for any of the classes. For the LSTM classifier, neither under-sampling nor over-sampling improved performance for the “defect” or “non-defect” classes. Although most of the under-sampling and over-sampling approaches did improve the performance of the LSTM classifier for the “possible defect” class, our similarity-based under-sampling methods did not outperform random under-sampling. Overall, the SVM classifier outperformed the NB and LSTM classifiers for the “defect” and “possible defect” classes, so we decided to pursue the SVM classifier that was trained on the original, imbalanced data.

**Table 1:** F_1_-scores (F) for “defect” (+), “possible defect” (?), and “non-defect” (−) tweet classes for three classifiers trained on the original, under-sampled, and over-sampled data sets

Classifier	Training set	F (+)	F (?)	F (−)
NB	Original, imbalanced training set (14,716)	0.54	0.44	0.94
NB	Under-sampling based on similar majority class tweets in original training set (5551)^a^	0.46	0.38	0.92
NB	Under-sampling based on similar false-negative majority class tweets (8015)^b^	0.44	0.40	0.92
NB	Random under-sampling control set (5551)^c^	0.50	0.43	0.93
NB	Random under-sampling control set (8015)^c^	0.51	0.44	0.93
NB	Over-sampling instances of minority classes with replacement (40,675)^d^	0.49	0.40	0.93
NB	SMOTE on original training set (39,148)^e^	0.36	0.30	0.95
SVM	Original, imbalanced training set (14,716)	0.62	0.52	0.96
SVM	Under-sampling based on similar majority class tweets in original training set (5551)^a^	0.62	0.43	0.96
SVM	Under-sampling based on similar false-negative majority class tweets (8015)^b^	0.58	0.51	0.95
SVM	Random under-sampling control set (5551)^c^	0.62	0.49	0.96
SVM	Random under-sampling control set (8015)^c^	0.62	0.50	0.96
SVM	Over-sampling instances of minority classes with replacement (40,675)^d^	0.62	0.46	0.95
SVM	SMOTE on original training set (39,148)^e^	0.62	0.51	0.96
LSTM	Original, imbalanced training set (14,716)	0.60	0.35	0.96
LSTM	Under-sampling based on similar majority class tweets in original training set (5551)^a^	0.55	0.33	0.91
LSTM	Under-sampling based on similar false-negative majority class tweets (8015)^b^	0.48	0.36	0.90
LSTM	Random under-sampling control set (5551)^c^	0.54	0.37	0.92
LSTM	Random under-sampling control (8015)^c^	0.59	0.45	0.95
LSTM	Over-sampling instances of minority classes with replacement (40,675)^d^	0.55	0.45	0.95

^a^Method (1) described in the “Methods” section

^b^Method (2) described in the “Methods” section

^c^Method (3) described in the “Methods” section

^d^Method (4) described in the “Methods” section

^e^Method (5) described in the “Methods” section

In addition to n-grams, we explored additional features for the SVM classifier. Table 2 presents the precision, recall, and F_1_-score of the classifier with the addition of word clusters and word/character lengths to n-gram features. Based on the default implementation of a paired *t*-test in Weka’s experiment environment, the additional features significantly improved the F_1_-score for the “defect” class (*P* < 0.05). The ablation study presented in Table 2 indicates, however, that word/character lengths did not contribute to the improved performance. Thus, word clusters, in particular, increased the classifier’s overall performance though, increasing recall at the expense of precision. Although the addition of word clusters significantly improved the classifier’s performance, the results of (1) using only word clusters or (2) leaving out n-grams significantly decreased the F_1_-score for all three classes (*P* < 0.05), indicating, perhaps unsurprisingly, that the classifier’s performance can be attributed primarily to the information provided by n-grams.

**Table 2:** Feature ablation for a support vector machine (SVM) classifier: precision (P), recall (R), and F_1_-scores (F) for “defect” (+), “possible defect” (?), and “non-defect” (−) tweet classes

Features	P (+)	R (+)	F (+)	P (?)	R (?)	F (?)	P (−)	R (−)	F (−)
All	0.62	0.68	0.65	0.58	0.45	0.51	0.96	0.96	0.96
- W/O word n-grams	0.22	0.55	0.32	0.43	0.22	0.29	0.94	0.89	0.92
- W/O word clusters	0.67	0.58	0.62	0.58	0.46	0.52	0.95	0.97	0.96
- W/O word/character lengths	0.62	0.68	0.65	0.57	0.45	0.51	0.96	0.96	0.96
Word n-grams	0.67	0.58	0.62	0.58	0.46	0.52	0.95	0.97	0.96
Word clusters	0.20	0.58	0.30	0.43	0.22	0.29	0.95	0.87	0.91

## Discussion

F_1_-scores of 0.65 for the “defect” (+) class and 0.51 for the “possible defect” (?) class represent a promising benchmark performance for automatic classification of highly imbalanced Twitter data.^25^ Given that birth defects are rare events and, thus, allow for some degree of manual analysis, we believe this performance provides a strong basis to facilitate cohort selection among the number of users identified via automatic classification. In general, a higher F_1_-score for the majority class is typical; in this case, it reflects that we have modeled the detection of birth defect outcomes for the data on which the classifier would be applied in practice. If we artificially balance the test set, the F_1_-scores for the SVM classifier improve to 0.76 for the “defect” class and 0.59 for the “possible defect” class. The comparative performance of the SVM and LSTM classifiers used in this study is consistent with our recent findings that SVM-based classifiers can outperform deep neural network-based classifiers for mining health-related events in imbalanced social media data.^26^ However, recent advances in language representation models^27^ may help improve the performance of deep neural networks for this task. Given that our under-sampling and over-sampling approaches generally did not improve performance for the LSTM classifier, we conclude that data-level approaches for addressing class imbalance with convolutional neural networks^28^ may not generalize to recurrent neural networks (e.g., LSTMs).

In order to demonstrate that automatic classification can be used to identify additional users for potential inclusion in our cohort, we implemented the SVM classifier in Python and deployed it on unlabeled tweets that were automatically collected^11^ since retrieving the tweets used for training and evaluating the classifiers in this study. Approximately 600 million additional tweets had been collected at the time we deployed the classifier, and our automatic rule-based retrieval^10^ detected 20,457 of them that mention birth defects, which were then preprocessed prior to automatic classification. The classifier predicted 678 tweets as “defect,” 359 tweets as “possible defect,” and 19,420 tweets as “non-defect.” Among the 7781 users who posted these 20,457 tweets, 414 users posted “defect” tweets, 185 users posted “possible defect” tweets (without also posting a “defect” tweet), and 7182 users posted “non-defect” tweets (without also posting a “defect” or “possible defect” tweet).

Among the 599 users who posted “defect” or “possible defect” tweets, 542 were new to our database—that is, not included among the 646 users whose timelines we analyzed to identify an initial cohort in our previous work.^10^ The addition of these users demonstrates that automatic classification indeed enables the long-term collection of social media data for large-scale epidemiological research of birth defects, including rare and more common ones. For example, given that congenital heart defects (CHDs) are the most prevalent birth defects reported among our initial cohort,^10^ social media data could soon enable a more thorough study of CHDs, which are also the most prevalent birth defects in the general population,^29^ are the leading cause of infant mortality due to birth defects,^30^ and have a largely unknown etiology.^31^

**Fig. 2:**
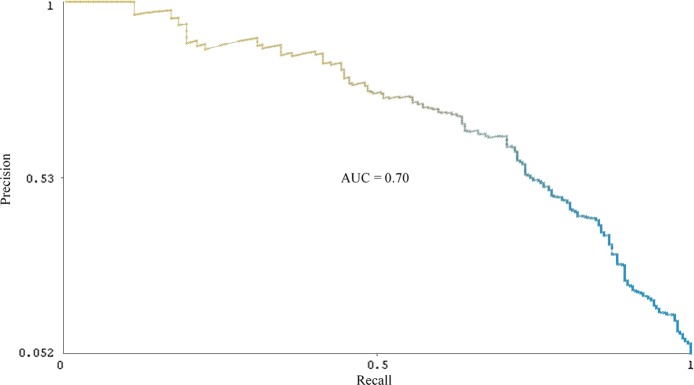
Precision-recall curve for a support vector machine (SVM) classifier for the “defect” tweet class

**Fig. 3:**
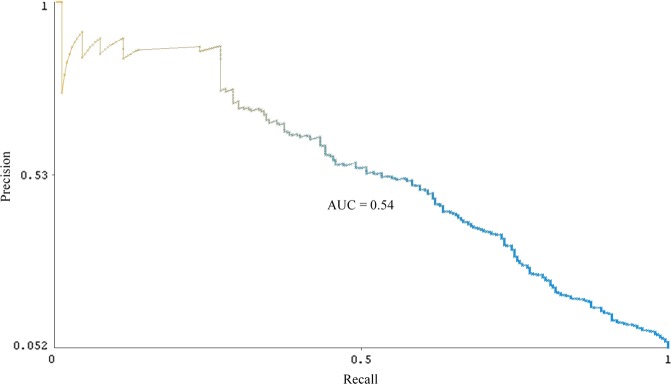
Precision-recall curve for a support vector machine (SVM) classifier for the “possible defect” tweet class

The identification of 542 additional users also demonstrates the utility of balancing precision (0.62) and recall (0.68) in tuning the classifier for the “defect” class. On the one hand, precision is important for automated cohort selection because only users actually reporting a birth defect outcome should be included among the cases for epidemiological analysis. For detecting cohorts reporting common events, such as pregnancy,^11^ a classifier can be optimized for precision and still be used to identify more than 100,000 users on social media. On the other hand, given that birth defects are rare events, optimizing the classifier for precision at the expense of recall would result in the automatic identification of very few users for inclusion in the cohort. However, considering that more than 7000 users were automatically identified as posting “non-defect” tweets, trading off precision for recall would require further reviewing an increasingly impractical number of users. Deploying the SVM classifier demonstrates that a balance of precision and recall can facilitate cohort selection for rare events on social media. Figures 2 and 3 provide the precision-recall curves for the “defect” and “possible defect” classes, respectively. (SVM classifiers do not provide probability estimates by default. To generate probability estimates for the precision-recall curves, we used an extension of the SVM classifier provided in the LibSVM library in Weka.^32^) The area under the curve is 0.70 for the “defect” class and 0.54 for the “possible defect” class.

Considering that automatic classification of three classes may be more difficult than two, especially since the “defect” and “possible defect” classes each represents only 5% of the annotated data, we performed an additional experiment in which the “defect” and “possible defect” tweets were collapsed into a single positive class. With two classes, the SVM classifier achieved a precision of 0.69, a recall of 0.60, and an F_1_-score of 0.64 for the positive class. More specifically, the number of true positives (i.e., “defect” or “possible defect” tweets correctly classified) increased from 272 to 289; the number of false positives (i.e., “non-defect” tweets classified as “defect” or “possible defect”) decreased from 149 to 129; and the number of false negatives (i.e., “defect” or “possible defect” tweets classified as “non-defect”) increased from 177 to 190. While the precision for both the “defect” and “possible defect” classes benefitted from collapsing the classes, the improved precision came at the expense of recall for the “defect” class. As shown in the confusion matrix in Table 3 and discussed in the error analysis that follows, the primary source of confusion for automatic classification of the three classes was false-negative “possible defect” tweets, and many of these errors seem to have been absorbed into the single positive class.

**Table 3:** Confusion matrix for a support vector machine (SVM) classifier for three classes of tweets: “defect” (+), “possible defect” (?), and “non-defect” (−)

	Predicted			
+	?	−		
163	12	64	+	
18	109	113	?	Actual
81	68	3974	−	

We conducted an error analysis of the SVM classifier trained on the original training set with n-grams, word clusters, and word/character lengths as features, focusing on false-negative “defect” and “possible defect” tweets in the test set of 4602 tweets—more specifically, the 177 “defect” and “possible defect” tweets misclassified as “non-defect.” Our analysis of the 113 false-negative “possible defect” tweets (from the 240 actual “possible defect” tweets in the test set)—the classifier’s primary confusion—reveals that the majority of them mention the name of a person. For example, in Table 4, 1 is annotated as “possible defect” because the name in the tweet is a personal deictic expression; that is, without additional contextual information (possibly supplied in the user’s timeline), the tweet is ambiguous as to whether the referent of the name is the user’s child. We attempted to model names as a generalized representation (i.e., substituting a name with “_name_”) in automatic preprocessing; however, our analysis reveals that, among the false-negative “possible defect” tweets that mention a name, more than half of the names were not detected in preprocessing and, thus, not normalized. Many of the other false-negative “possible defect” tweets similarly contain a personal deictic expression, such as *he* in 2 and *this little boy* in 3, or they omit an explicit lexical reference entirely, as in 4. Techniques for enhancing the representation of personal deixis—a characteristic feature of “possible defect” tweets—may improve the classifier’s recall for this class.

**Table 4:** Samples of false-negative “defect” (+) and “possible defect” (?) tweets, misclassified as “non-defect” (−) by a support vector machine (SVM) classifier trained on the original, imbalanced data set, with word n-grams, word clusters, and word/character lengths as features

	Tweet	Actual	Predicted
1	[name] was diagnosed with craniosynostosis and had surgery to repair it.	?	–
2	He has a cleft palate…..I don’t think he’s special needs.	?	–
3	I’m just in love w. this little boy. Hydrocephalus, split cerebellum, 2 vessel cord, dilated kidney, & survived	?	–
4	Born @ 25 weeks, hole in heart, no anal area, narrow airway, no vocal chords, floppy voicebox, missing vertebrae. Healed!	?	–
5	They couldn’t get a good picture of foot, and DR said it could be a Club Foot.	?	–
6	#DUPCoalition say the decision I was forced to make for my unborn daughter was wrong Trisomy 13, alobar holoprosencephaly,cystic hygroma	+	–
7	Her face #lovemygirl #downsyndrome #motheranddaughter	+	–
8	Raising a daughter with down syndrome makes me dream of a more inclusive society	+	–
9	My #clubfoot #cutie	+	–
10	We were given no options other than termination “We have no resources for you” #Trisomy18	+	–

**Fig. 4:**
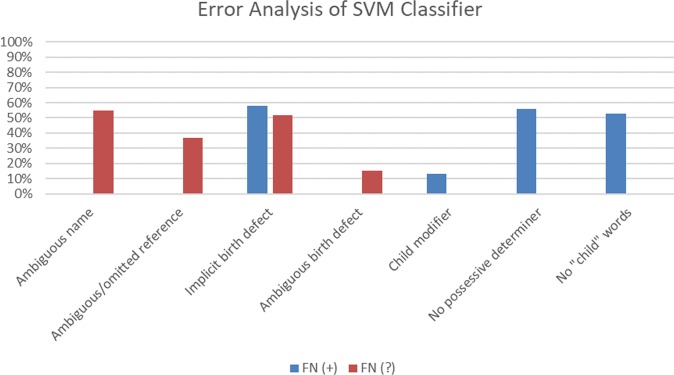
Possible sources of error for false-negative (FN) “defect” (+) and “possible defect” (?) tweets

Tweet 1 explicitly states that the person referred to by name has craniosynostosis (*was diagnosed with*), and 2 explicitly states that *he* has a cleft palate (*has*), but most of the false-negative “possible defect” tweets do not as explicitly encode this information in the text, as in 3 and 4. A human can reasonably infer that *this little boy* in 3 *has* hydrocephalus, and that the child implicitly referred to in 4 *was born with* a hole in her heart, but, without explicitly encoding this relationship between the person and the birth defect, the textual surface of the tweets may more closely resemble “non-defect” tweets that merely mention birth defects. Similarly, some of the false-negative “possible defect” tweets do not explicitly state that the child has a birth defect because, as in 5, they are ambiguous as to whether the child actually has the birth defect (*could be a club foot*). Figure 4 summarizes our analysis of errors for the false-negative “possible defect” and “defect” tweets in our held-out test set. As the proportions of errors indicate, many of the tweets contain multiple possible sources of error.

Most of the 64 false-negative “defect” tweets (from the 239 actual “defect” tweets in the test set) also imply having a birth defect. In addition, many of them, while referring to the user’s child, do not contain a common reference, such as *my child*. Some of the tweets, such as 6, explicitly refer to the user’s child (*my daughter*), but obscure this “defect” information by interrupting the pattern with a modifier *(my unborn daughter*). Many of the false negatives do not contain a possessive determiner (e.g., *my*, *our*) explicitly indicating that the user is referring to *her* child. Although 7 does contain the uninterrupted pattern, *my girl*, it is textually represented as a hashtag and, thus, modeled as part of a single token: *lovemygirl*. In 8, the first-person pronoun, *me*, suggests that the user is referring to her daughter, but *daughter* itself occurs with a determiner, *a*, that does not express a reference to the user’s child. Furthermore, many of the tweets do not use common “child” words. For example, 9 contains *my*, but the user refers to her child as *clubfoot cutie*. In 10, the user entirely omits an explicit reference to her child, though humans can infer that *termination* is a nominalization of the verb phrase, *terminating our child*.

Given the low prevalence of birth defects in the general population, some of the birth defects included in our rule-based approach to collecting tweets^10^ are rarely mentioned or not mentioned at all in the 22,999 tweets used for training and evaluating supervised classification. This underrepresentation could lead to overfitting the model to the birth defects mentioned more frequently in the annotated corpus. In our preprocessing, we made an effort to overcome the limited birth defects mentioned in the corpus by normalizing the span of the tweet that contains the birth defect. Without “hiding” the birth defects, the performance of the SVM classifier actually improves slightly to 0.68 for the “defect” class and 0.52 for the “possible defect” class; however, this improvement is likely the result of modeling particular birth defects as features and learning incidental associations with the classes. The classification performance reported in the “Results” section is intended to provide a more realistic representation of how the model would generalize in practice.

The tweet-level classification that we presented in this paper is the first step towards automatically identifying large cohorts of Twitter users whose pregnancies with birth defect outcomes could be observed via their publicly available tweets. To advance the large-scale use of social media as a complementary data source for studying birth defects, future work will focus on automating user-level analyses—in particular, (1) determining whether users identified as posting “possible defect” tweets have a child with a birth defect, and (2) verifying the availability of a user’s tweets during the pregnancy with a birth defect outcome. The first task would involve, for example, automatically resolving whether a personal deictic expression—for example, a person’s name or personal pronoun (e.g., *she*)—in a “possible defect” tweet is referring to the user’s child, based on contextual tweets in the user’s timelines. For the second task, we will further develop our system^33^ that automatically identifies a user’s prenatal period.

## Methods

### Data collection

To collect tweets for training and evaluating supervised machine learning algorithms, we first compiled a lexicon of birth defects (the Penn Social Media Lexicon of Birth Defects),^34–38^ which consists of ~725 single-word and multi-word terms. To enhance the retrieval of posts that mention birth defects on social media, we semi-automatically generated lexical variants of these terms (e.g., misspellings, inflections) using an automatic, data-centric spelling variant generator.^39^ Updated versions of the Penn Social Media Lexicon of Birth Defects (Supplementary Data 3) and its lexical variants (Supplementary Data 4) are available in the supplementary information files. To retrieve tweets containing (variants of) the terms, we implemented hand-crafted regular expressions for mining more than 400 million tweets, posted by more than 100,000 users, in our database.^11^ We post-processed the matching tweets by removing retweets and tweets containing user names and URLs that were matched by the regular expressions. Using the current version of the query, we have retrieved a total of 23,680 tweets, including the tweets retrieved for our feasibility study,^10^ which describes our data collection methods in more detail. This study received an exempt determination by the Institutional Review Board of the University of Pennsylvania, as it does not meet the definition of “human subject” according to 45 CRF § 46.102(f).

### Annotation

Two professional annotators annotated 23,680 tweets, posted by 7797 Twitter users, with overlapping annotations for 22,408 tweets. Annotation guidelines, available in Supplementary material of our related work,^10^ were developed to help the annotators distinguish “defect,” “possible defect,” and “non-defect” tweets. Table 4 includes examples of “defect” and “possible defect” tweets, and our feasibility study^10^ provides additional examples and a discussion of the three classes. Inter-annotator agreement was *κ* = 0.86 (Cohen’s kappa), considered “almost perfect agreement.”^40^ The tweets on which the annotators disagreed were excluded from the final data set, which consists of 22,999 annotated tweets: 1192 (5.12%) “defect” tweets, 1196 (5.20%) “possible defect” tweets, and 20,611 (89.67%) “non-defect” tweets. The “possible defect” class is a “placeholder” class indicating that analysis of the user’s timeline is needed for contextually determining if they are the mother of a child with a birth defect. Automating the analysis of user timelines, however, is beyond the scope of this paper and will be explored in future work. For now, we focus on the task of automatically classifying tweets, as described next.

### Classification

We used the data set of 22,999 annotated tweets in experiments to train and evaluate supervised machine learning algorithms. For the classifiers, we used (1) the default implementation of Multinomial Naïve Bayes (NB)^41^ in Weka 3.8.2, (2) the WLSVM Weka integration^42^ of the LibSVM^43^ implementation of Support Vector Machine (SVM), and (3) a Long Short-Term Memory Neural Network (LSTM),^44^ implemented in Keras running on top of TensorFlow. We split the data into 80% (training) and 20% (test) random sets, stratified based on the natural, imbalanced distribution of “defect,” “possible defect,” and “non-defect” tweets that would be automatically detected by the rule-based retrieval^10^ in practice. To optimize training, we further split the training set into 80% (training) and 20% (development) stratified random sets. Thus, our training set consists of 14,716 tweets, our development set consists of 3681 tweets, and our test set consists of 4602 tweets. In the remainder of this subsection, we will describe the data preprocessing, feature sets, classifier parameters, and data imbalance approaches used in supervised classification.

#### Preprocessing and features

For the NB and SVM classifiers, we performed standard text preprocessing by lowercasing and stemming^45^ the tweets. We also removed non-alphabetical characters (e.g., hashtags, ellipses, UTF-8 special characters), and normalized user names (i.e., strings beginning with “@”) as “_username_” and URLs as “_url_”. We did not remove stop words because, based on a preliminary feature evaluation using *information gain*,^46^ we found that commonly used stop words (e.g., *my*, *she*, *was*, *with*, *have*) occur in n-grams—contiguous sequences of *n* words—that are highly informative for distinguishing the three classes (e.g., *my baby*, *daughter with*, *she*, *was born*, *may have*). We also noticed in the feature ranking that some of the n-grams were semantically similar to n-grams that were not ranked as high (e.g., *our son*, *child with*, *he*). Given the context of this data set, we assumed that the ranking of these n-grams does not represent a meaningful semantic distinction but is merely a byproduct of the various linguistic ways in which users are referring to their children; thus, we reduced the semantic feature space by normalizing particular first-person pronouns (e.g., *my*, *our*) as “_fppron_”, references to a child (e.g., *son*, *daughter*, *child*) as “_child_”, and third-person pronouns (e.g., *she*, *he*) as “_tppron_”.

The information gain feature evaluation also suggested that, in the raw data, specific birth defect terms were informative linguistic features for distinguishing the classes. As Table 5 shows, such terms were mentioned markedly more frequently in “non-defect” tweets, suggesting that they tend to play a role in linguistically characterizing the “non-defect” class—an artifact of the imbalanced data set, but not an accurate representation of human decision making for a machine learning model. In addition, the birth defect terms in Table 5 represent some of the more frequently mentioned birth defects on Twitter, while most of the others are relatively sparse. In order to avoid overfitting the machine learning model to birth defects that are mentioned relatively frequently and tend to accompany “non-defect” tweets, we normalized the specific birth defect mentioned in the tweet—that is, the span of the tweet matched by the regular expressions in the lexicon-based retrieval^10^—as “_malformation_”.

**Table 5:** Examples and per-class frequencies of birth defect terms that distinguish the “defect” (+), “possible defect” (?), and “non-defect” (−) tweet classes in the raw annotated data

Birth defect term	+	?	−
CHD	115	79	914
Club foot	65	35	207
Down syndrome	407	467	11,157
Dwarfism	18	18	381
Gastroschisis	27	23	100
Hydrocephalus	22	39	256
Microcephaly	54	11	597
Trisomy 18	82	38	232

Finally, we also normalized people’s names as “_name_” in automatically preprocessing the tweets. During manual annotation, we found that, oftentimes, “possible defect” tweets are characterized by an ambiguous referent of a person’s name; that is, we do not know if the name refers to the user’s child. However, because of the heterogeneity of specific names across tweets, names would not be modeled as (part of) salient n-grams. To automatically detect names in the tweets, as a preliminary approach for experimentation, we compiled a lexicon, available in the supplementary information files (Supplementary Data 5), of the 200 most popular boys names and the top 200 girls names in the United States between 2010 and the present, identified by the Social Security Administration.^47^ Following preprocessing, for the NB and SVM classifiers, we converted the tweets to word vectors and used Weka’s default N-Gram Tokenizer to extract unigrams (*n* = 1), bigrams (*n* = 2), and trigrams (*n* = 3) as features.

In addition to n-grams, we explored word clusters as features for the SVM classifier. Word clusters provide generalized representations of semantically similar terms, in that terms appearing in similar collocate contexts, such as misspellings, are represented by the same cluster. The clusters are generated via a two-step process: first, vector representations of the words (i.e., word embeddings) are learned from large, unlabeled social media data, and then, the vectors are grouped via a standard clustering algorithm. We have found generalized representations via cluster numbers to be useful in our past work.^48^ For the present work, we used the Twitter word clusters made publicly available by Owoputi et al.^49^ Finally, we also used the tweets’ lengths in characters and words as features.

For the LSTM classifier, features were learned using the publicly available GloVe word vectors trained on 2 billion tweets.^50^ Any word in our corpus not found in the vocabulary of the pretrained vectors was represented with a zero vector and updated during training. We preprocessed the raw tweets in our corpus with the same operations and sequence used when creating the vectors. Specifically, we first normalized all user names (<USER>) and URLs (<URL>). Next, we inserted spaces before and after each slash character, and normalized all numbers (<NUMBER>). Then, we normalized all repetitions of the same punctuation (<REPEAT>) following the occurrence of the first one. Similarly, we deleted all letters repeated more than three times, and marked the occurrence of elongated words (<ELONG>). Finally, we replaced hashtag (#) characters (<HASHTAG>). We tokenized the tweets with the PTBTokenizer, and lowercased the tokens.

#### Classifier parameters

**Fig. 5:**
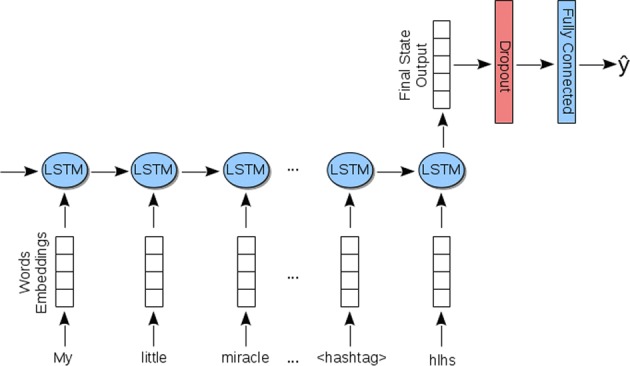
Architecture of the LSTM classifier

For the SVM classifier,^51^ we used the radial basis function (RBF) kernel. Upon tuning over the development set, we set the *cost* parameter at *c* = 100 and, to address the class imbalance problem, assigned higher weights to the minority classes. We scaled the feature vectors before applying the SVM for classification. For the LSTM classifier, we used 100-dimensional word embeddings pretrained on 2 billion tweets. The LSTM neural network consists of four layers sequentially connected: (1) the input layer that passes the word embedding vectors to the second layer, (2) an LSTM layer with 128 dimensions and a tanh activation function, (3) a dropout layer, and (4) a fully connected layer with three dimensions and a softmax function. We passed only the vector computed by the last hidden state of the LSTM layer to the third layer, ignoring the vectors computed by the other hidden states of the LSTM. Figure 5 illustrates the architecture of the LSTM classifier. We set the length of a sequence to 800 tokens and padded the right side of the sequence with zeros when it consisted of <800 tokens. We applied a dropout function with a probability of 0.5 during training in order to avoid overfitting. We trained the LSTM classifier for 15 iterations and fine-tuned the word embeddings during this stage.

#### Data-level approaches to class imbalance

In addition to assigning higher weights to the minority classes with the SVM classifier, we performed under-sampling of the majority (“non-defect”) class using several methods to address the class imbalance problem at the data level: (1) removal of majority class instances that were lexically similar to other instances in the training data, (2) removal of majority class instances that were lexically similar to minority class instances in the development set that were being misclassified as “non-defect” in the preliminary experiments, and (3) random under-sampling of the majority class. During annotation, we found a number of cases in which the same or a similar “non-defect” tweet was posted numerous times (e.g., fundraisers, news headlines, advertisements). Thus, our assumption underlying method (1) was that we could reduce the imbalance without losing linguistic information about the majority class. For method (2), we attempted to strategically remove “non-defect” tweets that were close to the linguistic boundary of the minority classes.

To compute lexical similarities between tweets, we employed the *Levenshtein ratio* (LR) method, which is calculated as ${\rm{LR}} \,=\, \frac{(\rm{lensum} \,-\, \rm{lendist})}{\rm{lensum}}$, where *lensum* is the sum of the lengths of the two strings, and *lendist* is the Levenshtein distance between the two strings. The equation results in a value between 0 (completely dissimilar strings) and 1 (identical strings). Levenshtein distance is a measure of the similarity between two strings computed as the number of deletions, insertions, or substitutions required to transform one string into another. When performing the under-sampling, we removed tweets that produced LR scores above a predefined threshold (*k*). We were able to choose the size of the under-sampled training sets by varying the value of *k*. We controlled methods (1) and (2)—the similarity-based under-sampling methods—by employing method (3)—random under-sampling of the majority class—based on the final size of the training sets for methods (1) and (2).

We also performed over-sampling of the minority (“defect” and “possible defect”) classes. We balanced the training set by (4) over-sampling the minority classes with replacement (i.e., multiplying the original sets of “defect” and “possible defect” tweets until they were nearly equal to the number of “non-defect” tweets), and (5) utilizing the Synthetic Minority Over-sampling Technique (SMOTE).^52^ With SMOTE, we nearly balanced the training set by creating artificial instances of “defect” and “possible defect” tweets in vector space. We used the default settings for the SMOTE filter in Weka. We performed all of the other over-sampling and under-sampling methods for all of classifiers, but applied SMOTE only for the NB and SVM classifiers.

### Reporting summary

Further information on research design is available in the Nature Research Reporting Summary linked to this article.

## Supplementary information

Supplementary Data 1.

Supplementary Data 2.

Supplementary Data 3.

Supplementary Data 4.

Supplementary Data 5.

Reporting Summary

## Data Availability

The annotated data that was used for training (Supplementary Data 1) and development (Supplementary Data 2) of machine learning algorithms in this study is available in the supplementary information files of this article. To download the tweets, a Python script is available at: https://bitbucket.org/pennhlp/twitter_data_download/src/master/.

## Code availability

The machine learning experiments were performed using open source software, Weka 3.8.2, following preprocessing in Python. The Python code that was subsequently prepared to train and evaluate the SVM classifier for deployment in this study is available at: https://bitbucket.org/pennhlp/birthdefectsclassifier/src/master/. The Penn Social Media Lexicon of Birth Defects (Supplementary Data 3), the lexical variants (Supplementary Data 4), and a file containing a list of 400 names (Supplementary Data 5), which are used for preprocessing in the Python script, are available in the supplementary information files of this article. The word clusters that are used as features in the SVM classifier are available at: http://www.cs.cmu.edu/~ark/TweetNLP/clusters/50mpaths2.
